# Formulation, Characterization, and* In Vitro* Evaluation of Transdermal Patches for Inhibiting Crystallization of Mefenamic Acid

**DOI:** 10.1155/2017/7358042

**Published:** 2017-11-12

**Authors:** Jirapornchai Suksaeree, Patsakorn Siripornpinyo, Somruethai Chaiprasit

**Affiliations:** Department of Pharmaceutical Chemistry, Faculty of Pharmacy, Rangsit University, Muang, Pathum Thani 12000, Thailand

## Abstract

The crystallization of mefenamic acid in transdermal patch is a major problem that makes the patch unstable and decreases the drug release. The additive was used to inhibit crystallization of a mefenamic acid. Among the different types of additives, polyvinylpyrrolidone (PVP) K30 and PVP K90 were studied and found to be highly effective in inhibiting the crystallization of the drug. The PVP presented as a solubilizer agent for mefenamic acid in matrix patches at the different ratio between drug : PVP, 1 : 2 and 1 : 2.5 for using PVP K30 and 1 : 1.5 and 1 : 2 for using PVP K90. The characterizations showed the homogeneous patches without the crystal form of the mefenamic acid in matrix patches. The release profiles of the mefenamic acid from the patches were investigated by Franz diffusion cells. Over the first 1 h, the release behavior of mefenamic acid from the patches obviously increased when PVP was used as a crystallization inhibitor. However, the ratio between drug : PVP K90 at 1 : 2 was found to be the most effective in increasing the drug release from patch. Thus, the PVP could be used as a crystallization inhibitor for mefenamic acid in matrix patches which will increase the drug release.

## 1. Introduction

Transdermal patches are an effective alternative route to deliver a small drug molecules through the skin into the systemic blood circulation and finally to the target organ [1, 2]. It will also be necessary for the delivered drug to reach its target sites and maintain a concentration at the target in therapeutic level [3, 4]. However, during this transport process, the drug can undergo severe biochemical degradations and the end products may ineffective and even toxic. Therefore, the drug substances that are used in their controlled release system should not easily degraded during administration and the drug can release as a plateau state in the range between the toxic level and the effective level [4–6]. The drug in matrix type patch has been increasing in popularity as effective transdermal delivery systems. The drug is dissolved or dispersed in the polymer matrix containing high concentrations of the drug are generally preferred and required that deliver therapeutic agents at a constant rate to the human body. The rate of the drug release from the matrix devices falls off with time, as the drug in the skin-contacting side of the matrix is depleted. Many classes of polymers such as cellulose derivatives, polyvinyl alcohol, carbopol, chitosan, and polyacrylates, have been used for transdermal patches [7, 8].

Mefenamic acid is a nonsteroidal anti-inflammatory drug and had a low solubility and high permeability which are classified in a Biopharmaceutics Classification System II. The powder of mefenamic acid can dissolve in water at 0.21 mg/mL but increasingly dissolved when some ethanol, propylene glycol, ethylene glycol, polyethylene glycol-400, glycerin, or 10% surfactant solutions are mixed as a cosolvent [9]. Mefenamic acid is used for relief of mild-to-moderate pain and primary dysmenorrhea by inhibition of cyclooxygenase-2 and prostaglandin synthesis. It is available for a tablets, capsules, and pediatric suspensions. It has a short elimination half–life at 2 hrs. Although oral administration is a popular route for mefenamic acid, it strongly requires frequent dosing every 6 hrs in order to maintain the steady-state plasma concentration [10]. This route is associated with gastrointestinal side effects such as ulceration, bleeding, or perforation of the stomach, small intestine, and large intestine, which can be fatal; therefore, it is contraindicated in patients with active ulceration or chronic inflammation of either the upper or the lower gastrointestinal tract [11]. Thus, transdermal administration is the one choice for delivering the mefenamic acid through the skin. This route can avoid the major gastrointestinal side effects, avoid the hepatic first-pass metabolism, and provide steady plasma levels by a single dose. The various transdermal dosage forms are developed such as nanoproniosomes [11], emulgel [12], and transdermal patch [13]. Moreover, the mefenamic acid is prepared by complexation reaction with monoethanolamine, diethanolamine, triethanolamine, and propanolamine, leading to enhancing the skin permeation rate through hairless rat skin [14].

However, the crystallization effect of the drug is a serious problem for the formulation design of the matrix transdermal patches [15, 16]. It makes the physicochemical properties instability of the patches, reduces the amount of drug release from the patches, and decreases the flux; it especially makes the patch lose its aesthetic appeal after crystallization [17, 18]. Thus, the crystallization inhibitor is studied and used to inhibit the crystallization of the drug. The effects of various additives (poloxamer 407, Tween 80, polyvinylpyrrolidone (PVP K30), and PEG-8 glyceryl caprylate/caprate) were investigated for the crystallization of ketoprofen in polyisobutylene adhesive matrix which is reported by Kim and Choi (2002). These various additives significantly increased the permeation rate of ketoprofen from polyisobutylene adhesive matrix during a study time of 120 days. The PVP K30 is found to be the most effective as a crystallization inhibitor of the ketoprofen in a matrix may significantly affect the efficacy and quality of the matrix transdermal patches [18]. Variankaval et al. (1999) reported a needle-like crystal and aggregates around the needles of estradiol in the transdermal patches when a drug has been dissolved in the polymeric adhesive patches [15]. Jain and Banga (2010) studied the various additives such as poloxamer 407, PVP K90, and copovidone for crystallization inhibition of captopril and levonorgestrel in the patches. The PVP K90 is the most effective additive in inhibiting the crystallization of the drugs [17]. In addition, the previous study found the crystallization of mefenamic acid in the transdermal patches which used ethyl cellulose and eudragit as a matrix film [19]. The PVP is interesting to be used as a crystallization inhibitor for mefenamic acid matrix patches. The preliminary study, the different types of additives (PVP K30 and PVP K90), and different ratio of drug : PVP are scanned and tested for the minimum concentration needed to inhibit crystallization by a simple mixing of the mefenamic acid drug and the additive in an appropriate solvent and looking for crystals under a microscope after the solvent is evaporated [20].

The objective of this study prepared the mefenamic acid matrix patches using different types of PVP as a crystallization inhibitor. The patches were made from ethyl cellulose as a matrix film and diethyl phthalate as a plasticizer. The drug and additive were dissolved in an appropriate solvent and mixed in the polymer solution, and then they were dried in hot air oven that produced the transparent mefenamic acid matrix patches. The mefenamic acid matrix patches were tested for their ability to inhibit crystallization by differential scanning calorimetry (DSC), X-ray diffraction (XRD), and scanning electron microscope (SEM). The release profile of mefenamic acid from all matrix type transdermal patches was studied by USP dissolution apparatus V.

## 2. Materials and Methods

### 2.1. Materials

Mefenamic acid (95% purity), ethyl cellulose, diethyl phthalate, PVP K30 average *M*_*w*_ 40,000, and PVP K90 average *M*_*w*_ 360,000 were obtained from Sigma-Aldrich (USA). The ethyl acetate and ethanol were of chemical grade.

### 2.2. Mefenamic Acid Matrix Patches Preparation

From the previous study, the ratios between drug to PVP in Table 1 were found to completely inhibit the crystallization of mefenamic acid [20]. Thus, these ratios were used to prepare the mefenamic acid matrix patches. The ethyl cellulose and diethyl phthalate were dissolved and mixed together in ethyl acetate. The PVP powder was completely dissolved in ethanol and, then, these solutions were homogeneously mixed by a mechanical stirrer. The composition of the mefenamic acid matrix patches containing an additive to inhibit crystallization is shown in Table 1. The mixture solution was sonicated for 30 min to reduce the air bubbles. Then, the mixture solution was poured into Petri-dish with area 70.88 cm^2^ and dried in hot air oven at 50 ± 2°C for 5 hrs. The dry mefenamic acid matrix patches were peeled-off from Petri-dish and kept in a desiccator until used for physical characterization and* in vitro *evaluation.

### 2.3. Physicochemical Characterization

#### 2.3.1. DSC Study

The DSC7 instrument (Perkin Elmer, USA) was used to determine the thermal behavior of the mefenamic acid matrix patches. The each mefenamic acid matrix patch was weighed about 10 mg into the DSC pan and hermetically sealed. The heating scan was 25°C to 400°C under a liquid nitrogen atmosphere with a heating rate of 10°C/min.

#### 2.3.2. XRD Study

The XRD instrument (model: X'Pert MPD, PHILIPS, Netherlands) was used to determine the crystallinity of the mefenamic acid matrix patches. The generator operating voltage and current of X-ray source were 40 kV and 45 mA, respectively, with an angular of 5–40°(2*θ*) and a stepped angle of 0.02° (2*θ*)/s.

#### 2.3.3. SEM Photography

The SEM5800LV instrument (model: JSM-5800 LV, JEOL, Japan) was used to study the surface morphology of the mefenamic acid matrix patches with high vacuum and a high voltage of 15.00 kV condition and using Everhart Thornley detector.

### 2.4. The Determination of Mefenamic Acid Content

The mefenamic acid content was determined by extraction technique with ethyl acetate and analyzed by the HPLC instrument. Each mefenamic acid matrix patch was cut into 1 cm × 1 cm squares and sonicated for 30 min. Samples were diluted 5 times with ethyl acetate and filtered using a 0.45 *μ*m cellulose acetate membrane. The mefenamic acid content in each sample was determined by comparison to the HPLC calibration curve.

### 2.5. *In Vitro* Release Evaluation

The release profile of mefenamic acid from all matrix type transdermal patches was studied by USP dissolution apparatus V using 900 mL of 2% w/v sodium dodecyl sulfate in distilled water as a receptor medium. The water bath was controlled at 37 ± 0.5°C while stirred constantly at 100 rpm. Five mL of the receptor medium was withdrawn at 0, 0.25, 0.5, 1, 2, 3, 4, 5, 6, 7, and 8 hrs and an equal volume of fresh receptor medium was immediately replaced. The mefenamic acid content in these samples was analyzed by the HPLC method. The experiments for each sample were performed in triplicate.

The kinetics for* in vitro *release of mefenamic acid were determined and calculated by (1) [21, 22]. The classical squared correlation coefficient (*r*^2^) was calculated from the slope of each linear portion plot.

(1) Qt=Q0+K0tlog⁡Qt=log⁡Q0+K1tQtQ0=KHt,

where

 
*K*_0_ is the zero constant rate (mg/h).
 
*K*_1_ is the first constant rate (mg/h).
 
*K*_*H*_ is the Higuchi's constant rate (mg/$\sqrt{h}$).
 
*Q*_*t*_ is the amount of mefenamic acid released (mg) in time *t* (h).
 
*Q*_0_ is the initial amount of mefenamic acid (mg) in the matrix type transdermal patches.

The HPLC analysis was performed by Agilent 1260 Infinity system (Agilent Technologies, USA). The mefenamic acid was eluted on reverse–phase ACE Generix5 C18 (4.6 mm × 150 mm, 5 *µ*m particle size, DV12-7219, USA) using methanol : acetate buffer pH 4.1 = 95 : 5 as a mobile phase with a flow rate of 1 mL/min and a 5-minute run time. The UV detector was set at 285 nm. The injection volume of sample was 10 *µ*L. The limit of detection was 1.10 *µ*g/mL and the limit of quantification was 3.33 *µ*g/mL. The HPLC method validation provided good linearity (*r*^2^ > 0.9997), accuracy, and precision in the required concentration range of mefenamic acid solution (Table 2).

## 3. Results and Discussion

Jain and Banga (2010) reported that the PVP is the most effective additive in inhibiting the crystallization of captopril and levonorgestrel using acrylate and silicone as an adhesive film [17]. From the previous study, we prepared the transdermal patches for mefenamic acid using ethyl cellulose and eudragit as a matrix film. It is found that the crystals of mefenamic acid dispersed in the patches [19]. Thus, PVP is interesting to be used as a crystallization inhibitor for inhibiting the crystallization of mefenamic acid. The different types of additive, PVP K30 and PVP K90, and different ratios of drug : PVP are scanned and tested for the minimum concentration used to inhibit crystallization by a simple method mixing of the mefenamic acid drug and the additive in an appropriate solvent. The crystals of mefenamic acid are determined under a microscope after the solvent is evaporated [20]. It was found that the appropriated ratio between drug : PVP used to inhibit crystallization of mefenamic acid is 1 : 2 and 1 : 2.5 for using PVP K30 as a crystallization inhibitor and 1 : 1.5 and 1 : 2 for using PVP K90 as a crystallization inhibitor. The mefenamic acid patches were cut into 1 cm × 1 cm specimens from five different positions and then the thickness of each specimen was measured and weighed. A thickness average was 218–253 *µ*m and a weight average was 62.71–82.12 mg (Table 1).

**Figure 1:**
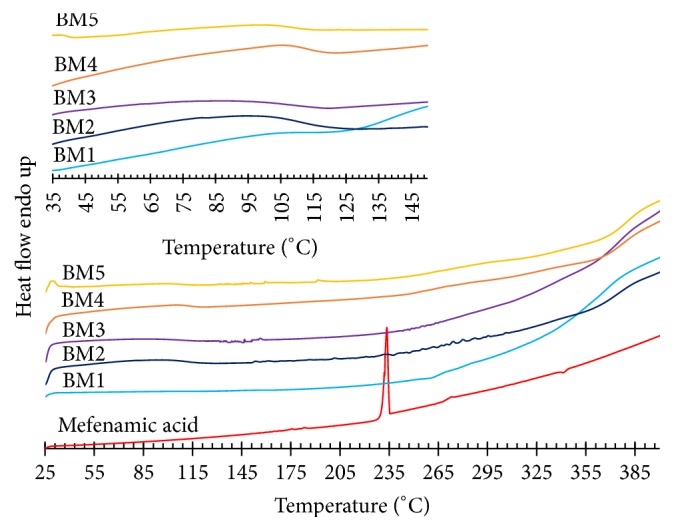
DSC thermogram of pure mefenamic acid and matrix type transdermal patches containing mefenamic acid.

Figure 1 shows the DSC thermogram of pure mefenamic acid and matrix type transdermal patches containing mefenamic acid. It was found that the melting point (*T*_*m*_) of mefenamic acid at 233.50°C related to the report of Cesur and Gokbel (2008) at 233°C [23]. The *T*_*m*_ of mefenamic acid was not found in all mefenamic acid matrix patches due to the minimal amount of mefenamic acid mixed in this matrix type transdermal patches. The ethyl cellulose, a water-insoluble polymer, had been formulated as a matrix film. The glass transition temperature (*T*_*g*_) was 130–133°C [24]. *T*_*g*_ is an important tool used to modify physical properties of drug and polymer molecules. *T*_*g*_ is shown by certain crystalline as well as amorphous solids [25, 26]. However, this work used the diethyl phthalate as a plasticizer. The plasticizer could insert between the polymer chains and spaces them apart from each other increasing the free volume. This results in polymer chains sliding past each other more easily. The polymer chains could move around at lower temperatures resulting in a decrease in *T*_*g*_ of a polymer. The properties of the polymer changed from those of a hard and brittle film to those associated with a soft and flexible film. As a result, diethyl phthalate was added to mefenamic acid matrix patches and *T*_*g*_ reduced to 85–115°C.

**Figure 2:**
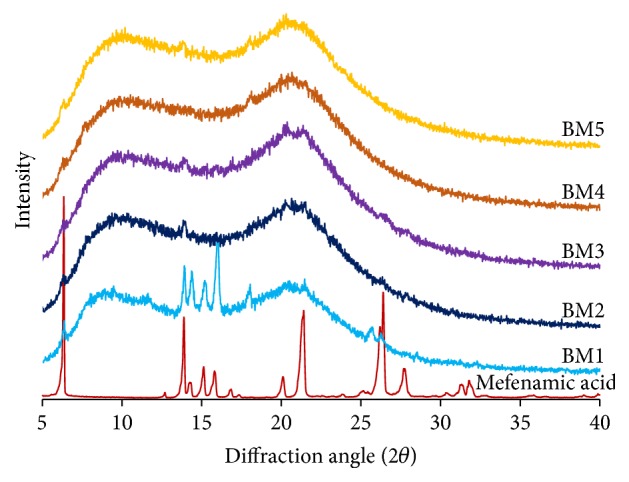
XRD patterns of pure mefenamic acid and matrix type transdermal patches containing mefenamic acid.

The crystal form of mefenamic acid in matrix transdermal patches was determined by XRD technique that is shown in Figure 2. The characteristic of crystal form of pure mefenamic acid was observed at 6.3°, 14.3°, 21.3°, and 26.3° (2*θ*) that coincided with those reported previously [23, 27]. This characteristic was found in BM1 formula that is a mefenamic acid matrix patch without PVP as a crystallization inhibitor. However, this characteristic was not found in BM2–BM5 formulas. Therefore, both different types of PVP K30 and PVP K90 were a suitable additive to completely inhibit the crystallization of mefenamic acid in matrix transdermal patches.

**Figure 3:**
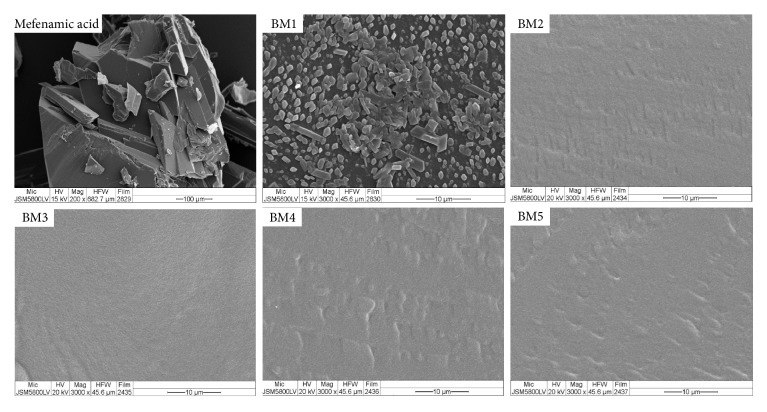
SEM photography of pure mefenamic acid and matrix type transdermal patches containing mefenamic acid (mefenamic acid and BM1 is represented from Suksaeree et al. 2017 [19]).

In Figure 3, the appearance of mefenamic acid crystals was as stick–shaped particles. The surface morphology of BM1 formula found the stick–shaped crystals of mefenamic acid which dispersed in this film. The surface morphology confirmed the compatibility of mefenamic acid with matrix films that obviously showed the homogeneous film after each PVP K30 or PVP K90 was mixed in those films. Thus, the inhibition of crystallization of mefenamic acid in matrix transdermal patches was a successful preparation by using PVP at the different ratio between drug : PVP, 1 : 2 and 1 : 2.5 for using PVP K30 as a crystallization inhibitor and 1 : 1.5 and 1 : 2 for using PVP K90 as a crystallization inhibitor.

Each sample of mefenamic acid matrix patch was cut into 1 cm × 1 cm squares, which were extracted in ethyl acetate by sonication method for 30 min. Then, they were analyzed for mefenamic acid content by HPLC method. The mefenamic acid content in matrix transdermal patches was 5.71 ± 0.45, 5.80 ± 0.53, 5.01 ± 0.29, 5.96 ± 0.52, and 5.87 ± 0.14 mg/cm^2^ for BM1, BM2, BM3, BM4, and BM5 formulas, respectively.

**Figure 4:**
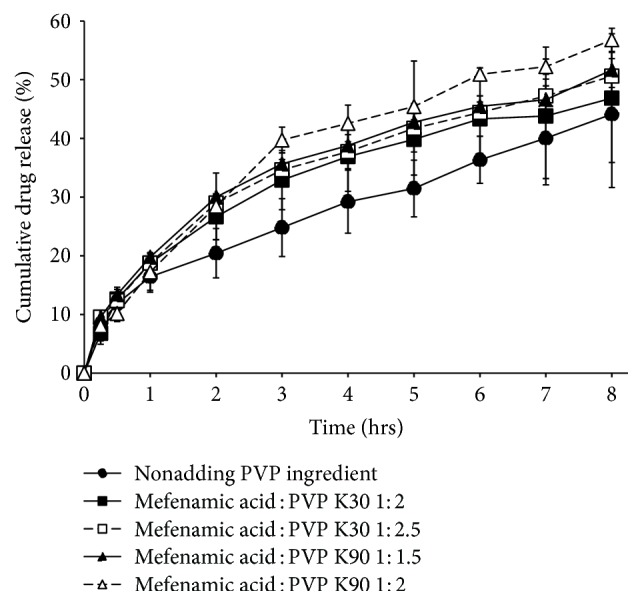
The percentage of cumulative drug release from matrix type transdermal patches.

The mefenamic acid could release 44.12  ± 12.48%, 46.85  ± 10.97%, 51.66  ± 12.99%, 53.66  ± 12.99%, and 56.79  ± 17.98% from BM1, BM2, BM3, BM4, and BM5 formulas, respectively (Figure 4). Although the ethyl cellulose is a semisynthetic cellulose derivative, hydrogen bonding capability between polymer and water molecules is relevant. There is polarity difference between the oxygen atom and ethyl group in the ethoxy molecule and, depending on the degree of substitution, hydroxyl groups are part of the cellulosic chain. Thus, there is potential to form a range of weak to strong hydrogen bonds between polymer and water molecules [28, 29]. PVP is the most hygroscopic polymer and can absorb a moisture content from an air environmental into its structure [30]. PVP could be mentioned to have acted just as a solubilizer for mefenamic acid in the matrix transdermal patches. Thus, BM2–BM5 formulas obviously increased the drug release compared to BM1 formula. The mefenamic acid could be released to a greater extent from matrix transdermal patches which increased the ratio of drug : PVP content in matrix transdermal patches. Using PVP K90 as a crystallization inhibitor showed the high percentage of drug release more than using PVP K30 as a crystallization inhibitor due to its high viscosity; it might be increasing the solubilization of the mefenamic acid in matrix transdermal patches.

The kinetics of mefenamic acid release were calculated as zero order, first order, and Higuchi's model that are shown in Table 3. Drug release in all matrix transdermal patches containing mefenamic acid was confirmed and also fitted to the Higuchi's model with high *r*^2^. The mefenamic acid release was determined by the diffusion control. This model was based on the hypotheses that (i) initial drug concentration in the matrix was much higher than drug solubility; (ii) drug diffusion takes place only in one dimension; (iii) drug particles were much smaller than system thickness; (iv) matrix swelling and dissolution were negligible; (v) drug diffusivity was constant; and (vi) perfect sink conditions were always attained in the release environment [19, 31].

## 4. Conclusions

The mefenamic acid patches were prepared by using ethyl cellulose as a matrix film and using the PVP as a crystallization inhibitor. A thickness and weight average of the patches were 218–253 *µ*m and 62.71–82.12 mg, respectively. As a result, the characterizations showed the homogeneous patches without the crystal form of the mefenamic acid drug, indicating completely achieved crystallization inhibition of mefenamic acid drug in the matrix patches. The release amount of mefenamic acid from the patches increased when PVP was used as a crystallization inhibitor and the ratio between drug : PVP increased. The PVP K90 was the high potential used as a crystallization inhibitor more than PVP K30. Drug release in all matrix transdermal patches was confirmed and also fitted to the Higuchi's model with high *r*^2^. In conclusion, the PVP acts as a crystallization inhibitor for mefenamic acid matrix patches which increasing the drug release from the patches.

## Figures and Tables

**Table 1:** The composition of mefenamic acid matrix patches.

Formulas	Drug : PVP	Ethyl cellulose (g)	Diethyl phthalate (g)	Ethyl acetate (mL)	Mefenamic acid (g)	PVP K30 (g)	PVP K90 (g)	Ethanol (mL)	Thickness (*µ*m)^a^	Weight (mg)^a^
BM1	—	1.50	0.45	40.00	0.38	—	—	—	239 ± 36	76.70 ± 7.48
BM2	1 : 2	1.50	0.68	40.00	0.38	0.76	—	10.00	253 ± 48	82.12 ± 12.91
BM3	1 : 2.5	1.50	0.74	40.00	0.38	0.95	—	10.00	218 ± 52	62.71 ± 10.72
BM4	1 : 1.5	1.50	0.62	40.00	0.38	—	0.57	10.00	248 ± 28	78.10 ± 9.18
BM5	1 : 2	1.50	0.68	40.00	0.38	—	0.76	10.00	228 ± 33	70.34 ± 14.82

^a^Measuring at five different positions on the patches.

**Table 2:** Method validation of mefenamic acid.

Concentration (*µ*g/mL)	Accuracy (% recovery)	Precision (% RSD)	
	Intraday	Intraday	Interday
10	103.59 ± 0.66	0.56	0.55
20	107.47 ± 1.53	1.33	0.38
30	104.90 ± 0.22	0.20	0.84

**Table 3:** Kinetic models for *in vitro *release of mefenamic acid.

Formula	Kinetic model	Equation	*r* ^2^
BM1	Zero order	*y* = 0.0808*x* + 0.1388	0.9455
	First order	*y* = −0.0264*x* + 0.1969	0.9648
	Higuchi's model	*y* = 0.2509*x* + 0.0091	0.9928

BM2	Zero order	*y* = 0.0602*x* + 0.1142	0.8849
	First order	*y* = − 0.0075*x* + 0.5662	0.8961
	Higuchi's model	*y* = 0.1930*x* + 0.0082	0.9879

BM3	Zero order	*y* = 0.0637*x* + 0.1206	0.9002
	First order	*y* = −0.0103*x* + 0.4605	0.9155
	Higuchi's model	*y* = 0.2029*x* + 0.0104	0.9930

BM4	Zero order	*y* = 0.0638*x* + 0.1280	0.8879
	First order	*y* = −0.0105*x* + 0.4522	0.9042
	Higuchi's model	*y* = 0.2042*x* + 0.0160	0.9894

BM5	Zero order	*y* = 0.0757*x* + 0.1065	0.9146
	First order	*y* = −0.0131*x* + 0.4418	0.9294
	Higuchi's model	*y* = 0.2386*x* − 0.0203	0.9872
